# *In vitro* approaches to mimic cardiac mechanical load dynamics for enhancing maturation and disease modelling

**DOI:** 10.1093/cvr/cvaf247

**Published:** 2025-11-20

**Authors:** Mariel Cano-Jorge, Sofia Gómez, Jaap den Toonder, Ye Wang, Robert Passier

**Affiliations:** Applied Stem Cell Technologies, Department of BioEngineering Technologies, Cardiovascular Health Technology Centre, TechMed Centre, University of Twente, 7500 AE Enschede, The Netherlands; Microsystems, Department of Mechanical Engineering, Eindhoven University of Technology, P.O. Box 513, 5600 MB Eindhoven, The Netherlands; Institute for Complex Molecular Systems, Eindhoven University of Technology, P.O. Box 513, 5600 MB Eindhoven, The Netherlands; Microsystems, Department of Mechanical Engineering, Eindhoven University of Technology, P.O. Box 513, 5600 MB Eindhoven, The Netherlands; Institute for Complex Molecular Systems, Eindhoven University of Technology, P.O. Box 513, 5600 MB Eindhoven, The Netherlands; Microsystems, Department of Mechanical Engineering, Eindhoven University of Technology, P.O. Box 513, 5600 MB Eindhoven, The Netherlands; Institute for Complex Molecular Systems, Eindhoven University of Technology, P.O. Box 513, 5600 MB Eindhoven, The Netherlands; Applied Stem Cell Technologies, Department of BioEngineering Technologies, Cardiovascular Health Technology Centre, TechMed Centre, University of Twente, 7500 AE Enschede, The Netherlands; Department of Anatomy and Embryology, Leiden University Medical Centre, 2300 RC Leiden, The Netherlands

**Keywords:** Mechanical load, Cardiomyocytes, Cardiac tissue engineering

## Abstract

The use of human pluripotent stem cells in cardiac tissue engineering has led to significant advances in the development of *in vitro* models of the human heart. However, full maturation of human pluripotent stem cell-derived cardiomyocytes has not been achieved. Current maturation strategies aim to replicate the native cardiac environment by incorporating the passive and active mechanical cues of the heart. Cardiac preload and afterload are key active mechanical loads that directly influence cardiomyocyte maturation and overall cardiac function. In this review, we explore the role of mechanical stimuli in cardiac development and cardiomyocyte maturation, with a focus on how preload and afterload dynamics can be replicated *in vitro* using platforms such as engineered heart tissues, stretchable membranes, bioactuators, engineered cardiac chambers, and microtissues. Additionally, we highlight the role of stimulation parameters used in dynamic preload modelling and how the incorporation of these active mechanical loads is applied in disease modelling.

## Graphical Abstract

**Graphical Abstract:**
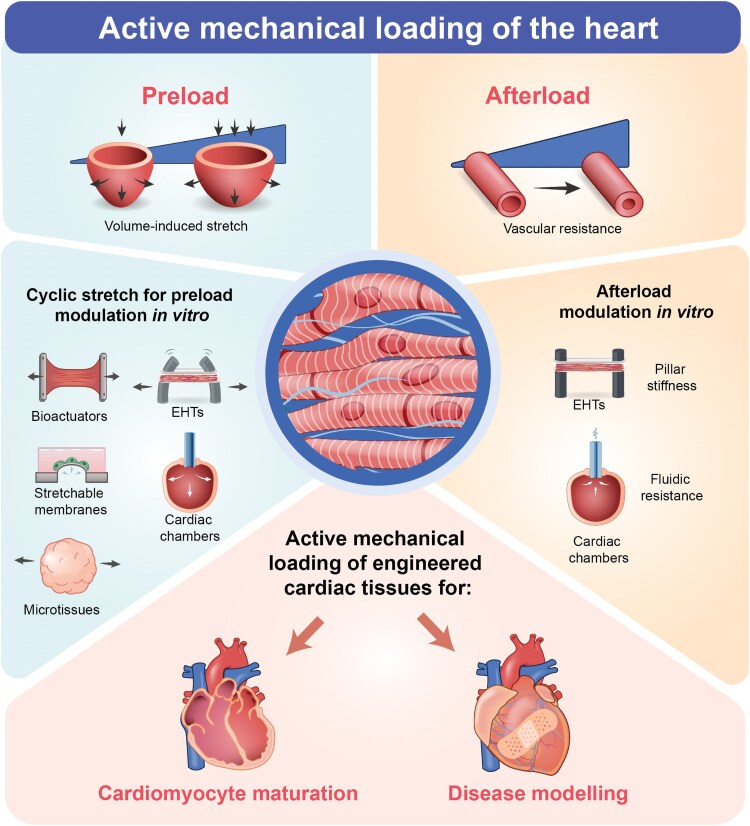

## 1. Introduction

Cardiac diseases represent a major healthcare burden worldwide, underscoring the need for more effective therapeutic strategies.^1^ In the past years, *in vitro* human induced pluripotent stem cell (hiPSC)-derived cardiac models have seen significant advancements, showcasing their potential as tools for disease modelling and drug testing.^2^ However, a remaining drawback of these models is the immature, foetal-like phenotype of hiPSC-cardiomyocytes (CMs). Various strategies have been explored to enhance hiPSC-CMs maturation, including three-dimensional co-cultures, incorporation of biochemical cues, and electrical pacing. More recently, mechanical stimulation has gained considerable attention as another maturation strategy due to its closer resemblance to *in vivo* heart dynamics.^3^

Throughout beating cycles, the heart is exposed to two major active mechanical loads: Preload and afterload. Preload is described as the extent of stretch the myocardium undergoes during diastole, whereas afterload is imposed by the vascular resistance the heart must overcome during systole. Recapitulation of these loads *in vitro* is crucial to improve the maturation and physiological relevance of hiPSC-derived cardiac tissues.

To replicate preload dynamics, various *in vitro* platforms have been developed to apply cyclic stretch to cardiac constructs, mimicking the degree of stretch experienced by cardiac muscle fibres during diastole due to blood filling. Although less explored, platforms modulating afterload dynamics have been developed by incorporating adaptable hydrodynamic or elastic resistances into cardiac tissue supports, mimicking vasculature resistance during systole.^4^ Although a positive impact on tissue structure and functionality^5^ has been shown, outcomes remain inconsistent, and differences in stimulation regimes across preload conditioning studies hamper our understanding of mechanically induced effects in cardiac tissues and CM maturation.

Advancing hiPSC-CM models requires a deeper understanding of the changes induced by mechanical stimuli both *in vivo* and *in vitro*. This review provides an in-depth exploration of the role mechanical stimuli play in cardiac development and maturation. By focusing on how CMs sense and respond to the mechanical properties of their environment, we highlight the importance of incorporating active mechanical loads into *in vitro* cardiac tissue models to enhance their physiological relevance. This review explores how preload and afterload can be modelled using platforms such as bioactuators, stretchable membranes, engineered heart tissues (EHTs), engineered cardiac chambers, and microtissues. Specifically, we discuss how different stimulation parameters of active preload can influence the maturation of hiPSC-CMs and enhance overall tissue performance. Finally, we highlight examples demonstrating the potential of incorporating active mechanical stimuli into *in vitro* models for disease modelling, thereby advancing our understanding of cardiac physiology and pathology.

## 2. Role of active mechanical cues in cardiac morphogenesis and function

The heart is the first functional organ formed during human development, essential for pumping blood to sustain the life of the developing embryo. This complex developmental process is partly mediated by the interplay of mechanical forces and transcriptional programs during four key stages: heart-tube formation, heart looping, trabeculation, and septation. First, a linear tube arising from the mesoderm is formed to supply blood to the embryo in a peristaltic fashion. As the tube elongates, it undergoes looping, establishing the primitive atrial and ventricular chambers. Next, the chamber walls thicken and form trabeculae that expand into the luminal space to facilitate the uptake of nutrients and oxygen.^6^ As the heart grows, septa extend from the compact myocardium towards specialized fibrous protrusions, establishing the four chambers of the heart and facilitating the formation of unidirectional valves.^7^

Throughout these morphogenesis stages, the heart is subjected to various mechanical stimuli such as shear stress, static and cyclic stretch, and vascular resistance. Mechano-transduction of these cues is crucial to ensure proper cardiac development and maturation. For instance, shear stress from blood flow is sensed by primary cilia in the endocardium, which triggers pathways that direct proliferation rates, differentiation, and growth of the endocardial and myocardial layers.^8^ Blood pressure within the heart generates a strain gradient along the myocardial wall that has shown to influence the morphology and proliferation rate of CMs, guiding the curvature, size, and proper formation of the primitive atrial and ventricular chambers.^9^ Similarly, the cyclic contractility of CMs has been shown to regulate the lateral fusion of sarcomeres during cardiogenesis and to influence cell migration during trabeculogenesis and septation.^10^ Other stromal cells, such as cardiac fibroblasts, respond to cyclic stretch by remodelling the extracellular matrix (ECM), increasing its stiffness to support CM organization.^11,12^ Lastly, external loading from vascular resistance is critical for proper heart looping and valve formation, underscoring the importance of mechanical stimuli in cardiogenesis.^13^

Mechanical cues also play a crucial role in regulating cardiac output. While heart rate is mainly governed by autonomic innervation and hormonal signals, stroke volume is influenced by cardiomyocyte contractility and mechanical constraints, such as cardiac preload and afterload. For instance, an increase in cardiac preload can trigger a short-term increase in systolic force, also known as the Frank–Starling mechanism.^14^ Similarly, physiological hypertrophic growth in response to increased afterload facilitates contractile force augmentation.^15^ Although these regulatory mechanisms allow the heart to fulfil haemodynamic requirements, long-term exposure to increased systolic or diastolic loads in the adult heart can also lead to maladaptive, pathological remodelling.

To mediate these responses to mechanical cues, CMs are equipped with several mechanosensory processes, including specialized structures within the sarcomeres, cell–cell junctions, focal adhesion complexes, and stretch-sensitive ion channels. For instance, sarcomeric proteins like titin and the MLP-TCAP–titin complex have been proposed to function as strain sensors within the sarcomere due to their molecular spring properties and anchoring sites to other cytoskeletal proteins.^16^ Similarly, intercellular contacts in intercalated discs have been reported to enable bidirectional mechanical force transmission between CMs. This is facilitated via adherens junctions and desmosomes that interconnect adjacent CMs with myofibrils and intermediate filaments, respectively.^17,18^ Mechanical cues propagate from CMs to the ECM, and vice versa, via costameres that link the ECM with the Z-lines of sarcomeres. This is mediated by integrin-talin interactions that form a molecular strain sensor through the stepwise unfolding of talin in response to strain.^17^ Lastly, stretch-sensitive ion channels such as TRP, Piezo1&2, and TREK1 also contribute to strain sensing by undergoing conformational changes in response to stretch, leading to enhanced influx of Ca^2+^ and K^+^, which can regulate cardiac contractility and activate pathways linked to regulating hypertrophic gene programs.^8,19^ Overall, these mechanisms enable sensing of active and passive aspects of the cardiac mechanical environment.

## 3. Biomechanics of the cardiac environment

**Figure 1:**
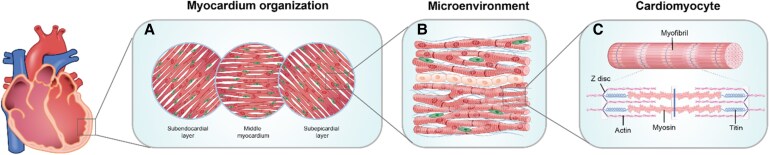
Schematic representation of the cardiac mechanostructure at different length scales. *(A*) The myocardium is organized in three different layers: subendocardial (*inner*), middle myocardium (*middle*), and the subepicardial (*outer*), illustrating the alignment of CMs (*elongated, red*). *(B*) At the tissue level, multicellularity of the myocardial environment is depicted, where CMs are anisotropically aligned in the ECM (blue fibers) and interact with other supporting niche cells, fibroblasts (*small, green*) and endothelial cells (*cuboid-shaped, orange*). *(C*) At the cellular level, CMs are shown with their organization into myofibrils, which contain the sarcomeric units composed of actin, myosin, titin, and Z discs—key components responsible for contraction and force generation.

**Figure 2:**
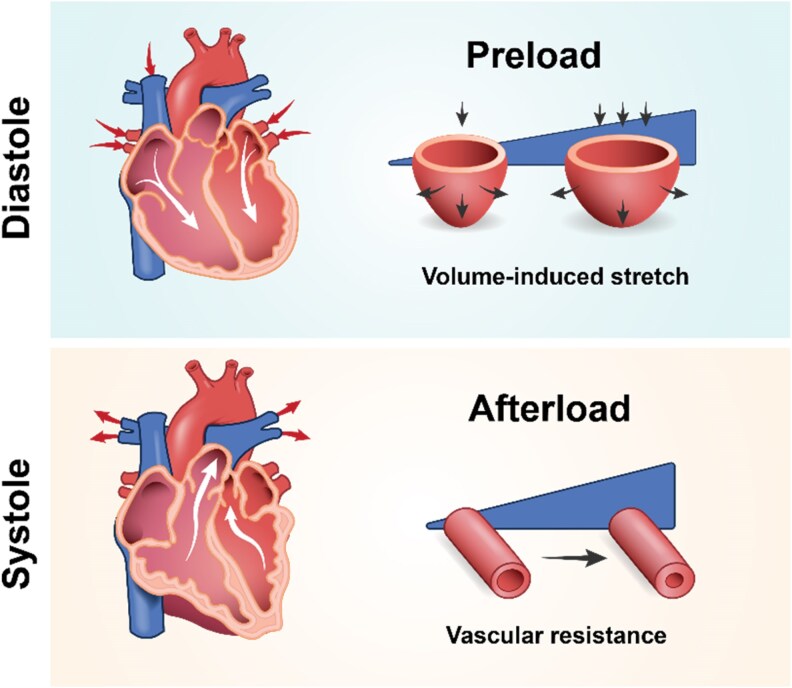
Schematic representation of haemodynamic loads in the heart. Cardiac preload represents the volume-induced stretch exerted on the heart during diastole, while afterload reflects the resistance the heart must overcome during systole for effective blood pumping. White arrows indicate the direction of blood flow.

The mechanical environment of the heart is highly complex, comprising both passive and active cues that influence cardiac output. Passive cues are provided by the intrinsic environmental architecture and composition of the heart, such as ECM stiffness and myofiber orientation (*Figure 1*). Meanwhile, active mechanical cues are linked to the dynamic nature of the cardiac cycle, with preload corresponding to volume-induced stretch and afterload introducing a pressure load the heart must overcome (*Figure 2*).

### 3.1 Cardiac mechanostructure

The intrinsic mechanical environment of the heart and its anisotropic structure are essential to ensure effective contraction and efficient blood pumping.^20^ For instance, the characteristic wringing and twisting motion of the heart during systole results from the highly organized myofibres orientation, which is essential for co-ordinated cardiomyocyte contraction, efficient force transmission, and optimal blood ejection.^21–23^ Specifically, the left ventricle (LV) consists of helically oriented myofibres having distinct angles between each layer (*Figure 1A*). This anisotropy of CMs and myofibres influences both the electrical and mechanical characteristics of the tissue.^24^

The cardiac ECM plays a crucial role in providing mechanical support and maintaining the mechanical integrity of the myocardium.^25^ Throughout development, the elastic modulus of the ECM progressively stiffens, transitioning from less than 10 kPa in the neonatal stage to approximately 25 kPa in the adult stage.^26^ This transition enables the heart to withstand the increasing mechanical forces experienced during the cardiac cycle as the heart develops, while providing structural support. In addition to ECM stiffening, viscoelastic responses also become more pronounced, facilitating the dissipation of mechanical energy and the adaptation to continuous mechanical stress.^27^ Mechanical properties of the ECM are modulated by growth factors and structural proteins, of which collagen is the most prominent.^28^ These collagen networks are essential for cardiac function, contraction, relaxation, and overall viscoelasticity of the myocardial tissue.^29^

In addition to structural proteins, cellular composition (*Figure 1B*) and intracellular components (*Figure 1C*) of CMs also influence the mechanical properties of the heart. Although CMs make up most of the myocardial volume, they represent only about 20–30% of the total cell number.^30^ The most prominent cell types in the heart are endothelial cells, constituting approximately 60% of the total, alongside smooth muscle cells that regulate vascular stiffness and flow.^31^ Fibroblasts, making up about 30%, are the second most predominant cells and play a crucial role in regulating and remodelling the ECM composition and the viscoelastic properties of the heart.^31,32^ The main protein responsible for modulating myocardial stiffness within the CMs is titin, a large sarcomeric filament that assists in the elastic recoil during diastolic filling.^33,34^ Microtubule networks within CMs also contribute by resisting compressive loads and absorbing energy from shear stress.^35,36^ These factors are essential for maintaining the mechanical properties necessary to ensure proper diastolic and systolic function.

By incorporating different aspects of the native myocardium *in vitro*, CMs exhibit improvements in structural maturity and elicit more physiological responses. For instance, culturing CMs on substrates that match *in vivo* myocardial elasticity has shown improved beating frequency and structural improvements.^37,38^ Similarly, incorporation of 3D models with crosslinked polymer networks such as GelMA allows for precise control of matrix elasticity and composition, while supporting cell–cell and cell–ECM interactions.^39^ Additionally, cardiomyocyte anisotropy has been recapitulated in *in vitro* models through incorporation of microgrooves, substrate topography changes,^21,40,41^ and scaffold architectures,^42–45^ which have shown to improve sarcomere organization and contractility.

### 3.2 Active mechanical loading of the heart

The dynamic mechanical environment of the heart is dictated by the external loading imposed by preload and afterload throughout the cardiac cycle (*Figure 2*). These active mechanical forces, mainly determined by blood return and vascular resistance, directly influence stroke volume. When preload or afterload experience changes—whether due to physiological conditions such as exercise or pathological states like hypertension—the heart initiates compensatory mechanisms, which are essential for maintaining a cardiac output that matches physiological demands.

#### 3.2.1 Preload

Preload is the tension generated in the muscle fibres of the myocardium as the ventricles fill with blood from the atria, stretching them just before contraction. This haemodynamic force corresponds to the ventricular end-diastolic volume, which directly modulates the contraction force of the heart. Preload also regulates stroke volume via the Frank–Starling mechanism. Its magnitude is influenced by several factors, including venous return, ventricular and atrial stiffness, and ventricular filling time.^46^ During preload, CMs undergo increased stretching, which lengthens sarcomeres and enhances the overlap between actin and myosin filaments, thereby optimizing their force-generating capacity. *In vitro* models often replicate the effects of myocardial stretch by manipulating tension within CMs. This tension can be induced by maintaining CMs at a fixed static length to promote tissue formation under anisotropic boundary conditions, or by actively stretching the CMs longitudinally to mimic preload changes.^47^

#### 3.2.2 Afterload

During systole, the pressure generated by ventricular contraction must exceed systemic vascular resistance to effectively eject blood into circulation. This resistance, known as afterload, corresponds to aortic pressure for the left ventricle and pulmonary artery pressure for the right ventricle.^30,46^ Increase in afterload requires the heart to generate larger contractile forces to overcome this resistance. Afterload influences cardiomyocyte shortening, enabling the heart to perform sufficient stroke against vascular resistance. Changes in afterload modulate sodium-calcium channels, resulting in elevated calcium levels in the sarcoplasmic reticulum and an increase in cardiomyocyte contractile force.^48^  *In vitro*, afterload is commonly modelled by adjusting the stiffness of the substrate or the pillars supporting cardiomyocyte culture and tissues. Additionally, afterload effects have been emulated by actively inducing transverse stretching of the cells.^49^

## 4. *In vitro* modulation of preload and afterload

**Figure 3:**
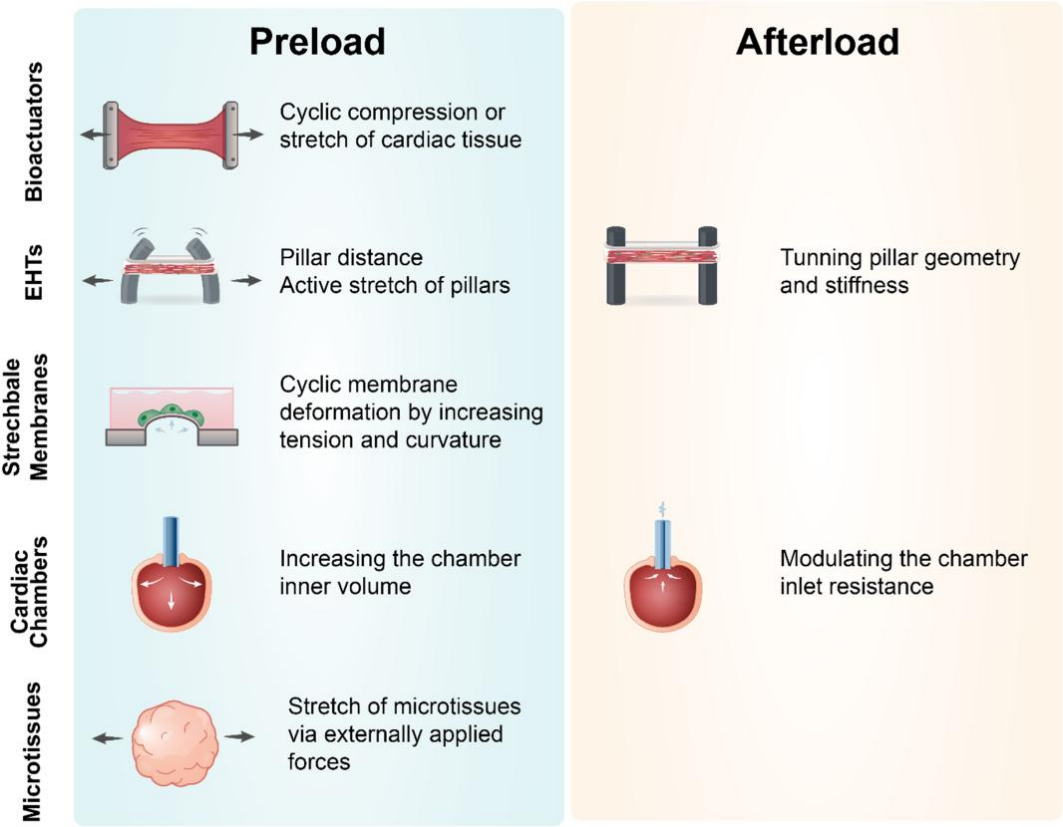
Schematic representation of the different *in vitro* approaches to model preload and afterload in cardiac tissues.

Engineered cardiac constructs have been widely used for *in vitro* research, and incorporating active preload and afterload to these constructs often requires the use of specialized mechanical supports or constraints. This underscores the need for tailored tissue platforms simultaneously allowing for tissue maintenance, mechanical load modulation, and readout acquisition. Here, we examine existing platforms such as bioactuators, stretchable-membranes, EHTs, engineered cardiac chambers, and microtissues, and how they can be used to modulate preload and/or afterload (*Figure 3*).

### 4.1 Bioactuators

**Figure 4:**
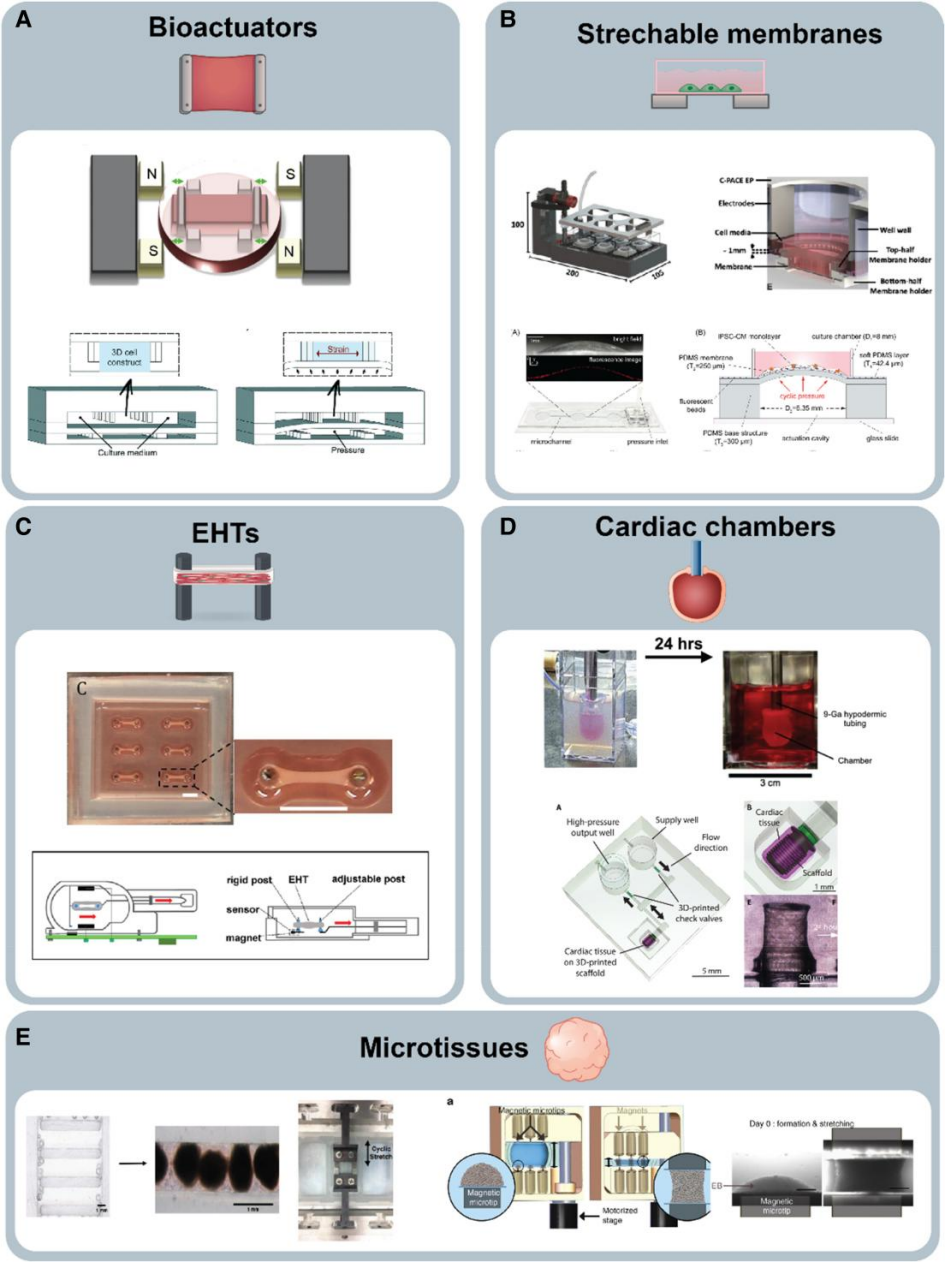
Overview of distinct *in vitro* systems exploiting dynamic mechanical stimulations. *(A*) Bioactuators utilize 3D cell constructs subjected to controlled cyclic strain through magnetic or mechanical forces, enabling studies on force generation and tissue mechanics. Reproduced with permission. ^50^Copyright 2015, Elsevier. ^51^Copyright 2016, Royal Society of Chemistry. *(B*) Stretchable membrane systems allow cyclic deformation of cardiomyocyte monolayers, using flexible membranes to simulate physiological strain, and study cellular responses. Reproduced under terms of the Creative Commons CC-BY 4.0 license. ^52^ Copyright 2020, Springer Nature. Reproduced with permission. ^53^ Copyright 2021, Elsevier. *(C*) Engineered heart tissues, or EHTs, involve 3D tissue constructs anchored between static or adjustable posts, facilitating the assessment of contractility, structural changes, and responses to mechanical load. Reproduced with permission. ^54^Copyright 2017, Elsevier. Reproduced under terms of the Creative Commons CC-BY 4.0 license.^55^ Copyright 2021, Ivyspring International Publisher. *(D*) Cardiac chambers mimic volumetric loading and pressure-driven mechanics, enabling modelling of chamber-specific functions, such as diastolic filling and systolic ejection. Reproduced with permission.^56^ Copyright 2018, Elsevier.^43^ Copyright 2022, The American Association for the Advancement of Science. *(E*) Microtissues, comprising cardiac organoids, spheroids, and embryoid bodies, stretched via actuation channels^57^ or magnetic forces.^58^  ^57^Copyright PLoS One, 2019. Reproduced under the terms of the Creative Commons CC-BY 4.0 license. ^58^Copyright Nature Communications, 2017. Reproduced under the terms of the Creative Commons CC-BY 4.0 license.

To provide a controlled environment for engineered cardiac constructs, different bioactuators, and customized heart-on-a-chip devices have been developed to incorporate mechanical stimuli. Most of these systems employ stretching mechanisms, achieved through linear motorized arms, electromagnetic forces, or pneumatic actuators. In these systems, preload is recapitulated through cyclic stretch of the cardiac tissues anchored onto the custom-made actuators (*Figure 4A*). For instance, human embryonic stem cell derived CMs were seeded onto gelfoam scaffolds anchored to a stretching apparatus, which exerted non-contact electromagnetic forces to displace the construct at both ends.^50^ Other systems have made use of microfluidic devices where hiPSC-CMs were cultured in hydrogels within a chamber enclosed by two PDMS layers, allowing for pneumatic actuators to cyclically compress the top layer of the chamber onto the construct.^51^ While these systems effectively mimic preload dynamics and support the maintenance of large cardiac constructs, replicating afterload effects remains a significant challenge that requires further development. Additionally, the lack of anisotropic tissue organization and direct contractile force acquisition limits the applications of these platforms.

### 4.2 Stretchable membranes

One of the earliest methods for applying mechanical stimulation to CMs involved the use of stretchable elastomeric membranes. In these systems, preload is recapitulated by stretching or bulging membranes under the CMs anchored on top (*Figure 4B*). Various custom-built systems^53,59^ and commercially available systems^60^ have been widely used to study the role of preload on cardiac maturation, with PDMS membranes being the most commonly used elastomeric material. These systems can vary in geometry, such as circular membranes or dog-bone shapes, to provide force distribution gradients across the constructs. Although these systems are typically limited to a monolayer, some have been adapted to support 3D constructs. This has been achieved with durable stainless steel supports to ensure anchoring of the constructs, while a PDMS membrane beneath provides preload stretching.^61^ A key advantage of these platforms is their ability to recapitulate multiaxial strain while requiring a relatively low number of cells, directly increasing experimental throughput. However, a major limitation is the difficulty in obtaining contractile force readouts. Furthermore, afterload modulation has not been effectively replicated yet in these systems, although some studies suggest that substrate stiffening could mimic afterload effects.^62^

### 4.3 Engineered heart tissues

Engineered heart tissues (EHTs) have been widely used as advanced *in vitro* cardiac platforms for drug discovery and disease modelling. These contractile tissue structures are made from cardiac cells embedded in a hydrogel, which is cast around two elastomeric pillars^63^ (*Figure 4C*). The deflection of the pillars upon tissue contraction facilitates the estimation of contraction force. In this configuration, the distance between the supporting pillars determines the static uniaxial tension experienced by the cells, mimicking cardiac preload and promoting anisotropic cell alignment. Additionally, geometrical and mechanical properties of the pillars, such as their stiffness, determine the static afterload that the EHTs must contract against. Recent efforts have focused on implementing active mechanical stimulation to EHTs. For instance, the incorporation of dynamic preload has been demonstrated in a study using an adjustable pillar whose distance from the remaining clamped support could be dynamically adjusted with a micromanipulator.^54,55^ Although more challenging, active control over the afterload imposed on EHTs has been demonstrated by tuning the effective stiffness of the supporting pillars with piezoelectric actuators coupled to a scaffold hosting the EHT. The versatility of EHTs, together with their accessible fabrication process and the ability to perform non-invasive contractile force readouts, makes them one of the most commonly utilized systems for cardiac tissue studies. However, their limitations lie in the restriction of cyclic strain to uniaxial deformation and the predominantly static afterload modulation they offer.

### 4.4 Cardiac chambers

Engineered cardiac chambers with pumping capability have recently emerged as novel models of the human heart. These models rely on the arrangement of CMs into hollow constructs enclosed around perfusion inlets, enabling fluid ejection upon synchronous tissue contraction^56,64,65^ (*Figure 4D*). The volume-handling capability of engineered cardiac chambers grants access to haemodynamic metrics such as pressure-volume loops, ejection fraction, and cardiac output, thus allowing for a more clinically relevant functional characterization.^65^ Notably, their structure and functional resemblance to cardiac compartments facilitate physiological mimicry of preload and afterload. In these models, preload is determined by the initial amount of fluid within the tissue prior to systole, which induces a multi-axial strain to CMs. Dynamic preload may be achieved through controlled fluid injection and ejection using pressure controllers or syringe pumps.^66^ Similar to vascular resistance, afterload is determined by the resistance at the perfusion inlet coupled to the chamber's lumen. Hence, the afterload imposed on the tissue may be tuned by using flow constrictors or by adjusting the dimensions of the perfusion inlet.^43^ Cardiac chamber models offer significant advantages in physiological relevance, as they simultaneously facilitate modulation of multiaxial preload and adjustable afterload while providing haemodynamic readouts. However, these models also present challenges, including the high cell numbers required, the labour-intensive fabrication process, and the need for specialized equipment to characterize tissue pump performance.

### 4.5 Microtissues

Microtissues, including cardiac organoids, spheroids, or embryoid bodies (EBs), are also widely used in cardiac research. Cardiac organoids, consisting of self-organized 3D multicellular structures derived from pluripotent stem cells, are used as *in vitro* models to study cardiac development.^67–69^ Spheroids, typically composed of aggregated CMs or mixed cardiac cell types, are valuable tools for studying cell–cell interactions, disease mechanisms, and drug responses.^70^ EBs are aggregates of stem cells that can be differentiated into cardiac lineages within the microtissue^71^ (*Figure 4E*). Due to the small, round shape (hundreds of µm in diameter) of microtissues, incorporation of preload dynamics through cyclic stretch is often challenging. However, mechanical loading has been achieved using external forces, either by embedding magnetic beads within the microtissues and actuating them with magnetic fields,^58^ or by confining them within deformable microchannels that allow controlled stretching of multiple microtissues simultaneously.^57^ Modulation of afterload in microtissues has not yet been demonstrated. Compared to other platforms, microtissues offer advantages such as high throughput and multicellular complexity, which makes them attractive for developmental studies, disease modelling, and drug screening. However, limitations include variability and restricted access to contractility readouts.

## 5. Parameters of active mechanical strain in preload modulation and their roles *in vivo*

When applying active mechanical stimulation to mimic *in vivo* preload dynamics, several key parameters must be considered. While these parameters and their corresponding actuation ranges are often based on *in vivo* values, they do not always directly translate to *in vitro* systems. Moreover, each platform requires precise strain calibrations and characterization to ensure that the appropriate stimulation parameters are applied to the samples. In this context, we will further explore the role of these parameters and the comparison with their *in vivo* counterparts.

### 5.1 Frequency

The cardiac beating rate or frequency serves as an indicator of developmental stages and physiological status. During development, the beating frequency increases from the heart tube stage to the four-chambered stage, peaking at around 170 bpm, and then decreases to around 130 bpm as the heart transitions from the late foetal stage to the neonatal stage. From the neonatal to adult stage, the heart rate stabilizes at about 60 bpm at rest but can elevate to approximately 180 bpm during peak exercise.^72^

The most commonly employed frequency for *in vitro* models with active mechanical stimulation typically ranges between 1 and 1.25 Hz, reflecting the resting heart rate in adults.^73^ Integrating this frequency within an *in vitro* stretching model more accurately replicates the mechanical conditions of the heart. Isolated CMs or stem cell-derived CMs often exhibit spontaneous beating, due to their depolarized resting potential and lack of defined and organized sarcomeres observed in adult CMs.^74^ Although synchronizing external stretch with the intrinsic beating can be challenging, some studies have employed sensing techniques to match the imposed stretching period to the tissue's relaxation phase.^75^ Other approaches have applied stretch at fixed frequencies and phases that do not necessarily match with the tissue intrinsic beating rhythm, thereby allowing spontaneous beating of CMs to synchronize to the stimulus.^76^ From these approaches, we can observe CM's capacity for long-term modifications, underscoring the importance of carefully selecting the stimulated frequency.

### 5.2 Strain

Throughout the diastolic and systolic phases of the cardiac cycle, pulsatile changes manifest at the cellular level as cyclic strain and stress.^77^ During diastole, cardiomyocytes (CMs) undergo cyclic stretching that generates tensile strain along the longitudinal, radial, and circumferential axes, with diastolic wall strain reaching ∼40% in healthy adults.^78^ Various experimental systems can induce these diastolic strain variations *in vitro*. EHTs mimic longitudinal strain, while engineered cardiac chambers and stretchable membranes facilitate multiaxial stretch modelling, which better represents the wall tension profiles experienced in hollow organs during filling pressures.

Strain is experimentally simpler to measure than stress, as it involves applying a controlled mechanical force and directly observing tissue deformation. Stress measurement, in contrast, can be determined by applying a known force to specific regions of the tissue and observing the resulting deformation, which allows for estimating the force per unit of area. Alternatively, the stress can be estimated from measured strains, provided the elastic modulus of the stimulated tissue is known. In experimental systems, strain amplitude indicates the maximum deformation imposed on cells and tissues. In this regard, early work by Fink *et al*. showed that stretches between 4 and 20% produced higher contractile forces in neonatal rat CMs,^79^ findings later reproduced by multiple studies and confirming cyclic strain's importance in cardiomyocyte maturation.^5^

In contrast to diastolic strain (preload), systolic strain refers to the compressive deformation imposed by the tissue on itself as it shortens during active contraction. *In vivo*, peak systolic strain ranges from 15–20% in longitudinal and circumferential directions and from 30–45% in the radial direction.^80,81^  *In vitro,* the magnitude of systolic strain is influenced not only by the contractile force of the tissue but also by the afterload, which is determined by the mechanical properties of its anchoring materials. For instance, the elastic nature of supporting pillars in EHTs allows the tissues to actively shorten during contraction, resulting in a significant systolic strain.^63^ Similarly, engineered cardiac chambers often exhibit a significant systolic strain through active contraction. However, this strain may be limited when the tissue is mechanically constrained by stiff scaffolds or when increased afterload is imposed via flow restriction, depending on the tissue's contractile capacity.^43^ In contrast, platforms like stretchable membranes or bioactuators, which are often anchored to rigid, non-compliant supports, exhibit minimal tissue shortening, resulting in negligible systolic strain. While systolic strain may promote contractile performance and support tissue maturation, *in vitro* diastolic strain is often considered more critical for mimicking physiological cardiac preload effects.^5^

### 5.3 Pattern

The heart is continuously subjected to cyclic preload. However, for *in vitro* recapitulation, the active load applied throughout a stimulation protocol can vary over time. Hence, three distinct stimulation patterns have been defined. In continuous stimulation, a cyclic strain of a fixed amplitude is consistently imposed on the tissue. In intermittent patterns, an active cyclic strain is applied for a few hours each day over a series of days or weeks. Lastly, in progressive patterns, a static strain is imposed on a tissue and gradually increased in amplitude at daily or multi-day intervals.

Discerning the differential effects among these patterns remains a challenge. However, some studies suggest that intermittent cyclic stimulation may enhance tissue performance compared to continuous stimulation.^82^ Although intermittent stimulation does not fully replicate physiological conditions, introducing resting periods between stimulation series can prevent mechanical overloading, allow for matrix remodelling, and improve tissue properties. For instance, studies on engineered 3D cardiac constructs made from neonatal rat CMs have demonstrated that intermittent compressive stimulation enhances sarcomere striation and increases FGF release relative to continuous stimulation.^83^ Similarly, engineered heart valve tissue constructs made from human venous myofibroblasts subjected to intermittent cyclic stretch exhibited enhanced collagen fibre alignment and higher tissue modulus compared to continuously stimulated constructs.^82^ However, there are no studies reported that confirm these findings in iPSC-derived cardiac constructs.

In contrast, progressive stimulation protocols differ fundamentally by gradually increasing the mechanical load over time, simulating the adaptive increase in blood return that accompanies the growth of the organism. This approach has yielded promising results, particularly in EHTs, where progressive mechanical stimulation led to significant enhancements in contractile performance and physiological hypertrophic growth of hiPSC-CMs,^53^ a feature not commonly observed with intermittent or continuous protocols.

### 5.4 Duration

The duration of mechanical stimulation protocols for *in vitro* cardiac tissues typically depends on the specific stimulation pattern. For instance, continuous stimulation protocols commonly range from 2 to 8 days, whereas intermittent stimulation protocols, where load is applied for only 30 min to a few hours per day, often span longer durations from 1 to 4 weeks. Progressive stimulation protocols generally last 2–3 weeks, to accommodate a gradual increase in mechanical loads.

The optimal duration required to achieve sustained maturation effects or improvements in tissue performance remains uncertain. However, several studies have demonstrated that cyclic mechanical stimulation induces time-dependent responses in gene expression. For instance, Rysa *et al*., evaluated the effects of cyclic stretch on neonatal rat cardiomyocytes at 1, 4, 12, and 24 h of duration. They observed that immediately early genes, such as *c-fos*, were up-regulated as early as 1 h into stimulation, and that the number of differentially expressed genes increased proportionally to the duration of stretch. During the acute phase (1–12 h), they observed a significant upregulation in the NRF2-mediated oxidative stress response canonical pathway, whereas in the long-term phase (24–48 h), inflammatory pathways showed the highest levels of activation. These results are consistent with a similar study by Cheng Chen *et al*., which subjected human ventricular CMs to various durations of continuous cyclic stretch.^84^ In this study, short-term stimulation (1–12 h) resulted in enrichment of lipid metabolism pathways whereas longer durations of stimulation (24–48 h) led to the up-regulation of fibrosis and inflammatory pathways.^84^ Nevertheless, extending stimulation over days or weeks is often necessary to achieve functional improvements in contractile performance, due to the slow dynamics of myofibrillogenesis and sarcomere reassembly in response to mechanical strain.^85^

### 5.5 Duty cycle

The human heart naturally exhibits an asymmetric duty cycle, where approximately one-third of the cardiac cycle corresponds to systole and the remaining two-thirds to diastole. While anatomical features can influence this asymmetry, intrinsic cardiomyocyte contraction is the primary driver, ensuring sufficient time for ventricular filling.^86^ Although less explored, the duty cycle of strain pulses is an important parameter in both continuous and intermittent stimulation protocols. Most studies employ sinusoidal^87^ or rectangular^75^ waveforms with equal loading and unloading phases. However, some protocols have incorporated asymmetric duty cycles, where the duration of the active load differs from the unloading period. For instance, studies have explored duty cycles where 12.5% or 37% of the cycle time is assigned to stretch and the remainder to relaxation.^50,75^ While incorporating such asymmetry is of interest, a longer stretch period may offer better physiological resemblance.

## 6. Active preload modulation promotes cardiomyocyte maturation

Incorporation of active preload mechanical stimulation in *in vitro* models of cardiac models induces a range of phenotypical effects that contribute to overall tissue performance. In this section, we examine how different stimulation regimes (*Table 1*) impact functional and structural characteristics of cardiac tissues. It is important to note, however, that research into the effects of afterload stimulation is not included in this analysis due to the limited availability of studies and the complexity of implementing this form of stimulus *in vitro*.

**Table 1:** Overview of experimental parameters used in *in vitro* models employing active preload stimulation

	Ref	Platform	Cell type	Frequency	Strain	Duty cycle	Duration	Pattern
**2D Model**	Chun 2015^88^	Stretchable membrane	hiPSC-CM	1 Hz	5%	50% duty cycle	2 days	Continuous
	Kroll 2017^89^	Stretchable membrane	hiPSC-CM	1 Hz	5%	50% duty cycle (16% expansion, 18% hold phase, 16% contraction)	3–7 days	Continuous
	Cortes 2020^52^	Stretchable membrane	hiPSC-CM	1.33 Hz	2.34%		2, 4 or 7 days	Continuous
	Dou 2021^53^	Stretchable membrane	hiPSC-CM	1 Hz	5%, 10%, 15%, 20%		8 days	Continuous
	Song 2022^90^	Stretchable membrane	hiPSC-CM	1 Hz	10%		2 days	Continuous
**3D Model**	Marsano 2016^51^	Bioactuators	hiPSC-CM	1 Hz	10%		5 days	Continuous
	Tulloch 2011^91^	Bioactuators	hESC-CM/hiPSC-CM	1 Hz	5%	Sinusoidal	4 days	Continuous
	Mihic 2014^50^	Bioactuators	hESC-CM	1.25 Hz	12%	37% stretching, 63% relaxation	3 days	Continuous
	Zhang 2017^54^	EHT	hESC-CM/hMSCs/hFBs	1 Hz	10% preload and 10% strain		3 days	Continuous
	Jayne 2021^75^	Bioactuators	iPSC-CM/hMSCs	0.5 Hz	1.1%	Rectangular, 12.5% duty cycle	1–5 min	Continuous
	Lu 2021^55^	EHT	hiPSC-CM	NA	6% increase per day	NA	21 days	Progressive
	Kensah 2013^92^	Bioactuators	hESC-CM	NA	6.6% increase at days 4, 10, 14 and 17	NA	21 days	Progressive
	Ruan 2015^93^	Bioactuators	hESC-CM	1 Hz	5%		14 days	Continuous
	LaBarge 2019^57^	Microtissues	hiPSC-CMs	1 Hz	10%		7 days	Continuous

EHT refers to engineered heart tissue; hiPSC-CM, human induced pluripotent stem cell-derived cardiomyocyte

;hESC-CM, human embryonic stem cell-derived cardiomyocyte; hFBs, human dermal fibroblasts; iPSC-CM, induced pluripotent stem cell-derived cardiomyocyte; NA, not applicable.

### 6.1 Structural

Enhancements in CM maturation are often evident through structural improvements such as cell alignment, elongation, and the development of organized sarcomeres. These advancements are essential for key physiological functions, including efficient contraction and electromechanical coupling.^94,95^ Recent studies have shown to partially replicate these structural enhancements through the incorporation of dynamic preload, where differences between monolayer and 3D tissues have been observed (*Table 2*). For instance, regardless of the stimulation regimes applied to CM monolayers on stretchable membranes, no significant changes in CM elongation have been reported. In contrast, elongated morphology and evidence of Z-band formation have been noted in hiPSC-CMs^51,55^ and hESC-CMs^50^ from 3D-tissues cyclically stretched with bioactuator and EHT platforms. However, signs of mechanically induced alignment have been observed in both monolayer and 3D cardiac tissue models. Studies have demonstrated that hiPSC-CM monolayers subjected to isotropic mechanical strain using circular stretchable membranes tend to orient radially to the strain direction^53^ and to display increasingly pronounced alignment as strain levels reach up to 15%. Conversely, when CM monolayers are exposed to anisotropic cyclic strain using rectangular stretchable membranes, they align along the direction of the strain.^90^ For the stimulation of 3D models, CM alignment has been reported to have an overall 1.3 to 2.0-fold increase under varying stimulation regimes. Specifically, EHT systems, constrained by two supports, have demonstrated enhanced alignment due to their anisotropic mechanical stimulus. However, some studies suggest that in 3D tissue models, cyclic stretch does not significantly enhance CM alignment beyond what is achieved with static strain.^54,91^

**Table 2:** Overview of structural enhancements in *in vitro* cardiac tissues subjected to active preload stimulation

Ref	Platform	Structure				
		Morphology	Alignment	Sarcomere length	Protein expression	Hypertrophy
Chun 2015^88^	SM				↑Cx43	
Kroll 2017^89^	SM	NC	No	1.55 µm (↓ 0.86-fold)	↑ N-cadherin	
Cortes 2020^52^	SM	NC		1.90 µm (No change)	↓ Cx43 ↑ α-actin	
Dou 2021^53^	SM		↑ n.q.	5%: 1.806 µm (↑ 1.03 fold) 10%: 1.890 (↑1.08-fold) 15%: 1.908 (↑ 1.09-fold) 20%: 1.911 µm (↑ 1.09-fold)		
Song 2022^90^	SM		↑ n.q.	1.794 µm (↑ 1.08-fold)	↑ MLC2v NC in Cx43	↑ ANP ↑ BNP
Marsano 2016^51^	BA	↑ Elongation (n.q)			↑Cx43	
Tulloch 2011^91^	BA		↑ 2-fold			
Mihic 2014^50^	BA	↑ Elongation (n.q)			↑Cx43 ↑ α-actin ↑ cTnT, ↑ MLC2v ↓ MEF-2C	↑ ANP
Zhang 2017^54^	EHT		↑ 1.5-fold	1.8 µm (↑1.17-fold)		
Jayne 2021^75^	BA					
Lu 2021^55^	EHT	↑ CM volume (2-fold)	↑ 1.33-fold	2.19 µm (↑1.17-fold)		↑ CM size
Kensah 2013^92^	BA		↑ n.q.			
Ruan 2015^93^	BA		No		↑Cx43, ↑ cTNT	
LaBarge 2019^57^	MT				↑Cx43, ↑ cTNI, ↑ N-cadherin	

Upward and downward arrows indicate increases or decreases of the markers, respectively.

ANP: Atrial Natriuretic Peptide; BNP: B-type Natriuretic Peptide; BA: bioactuators; CM: cardiomyocyte; Cx43: connexin 43; cTnT: Cardiac Troponin T; EHT: engineered heart tissue; MEF-2C: Myocyte Enhancer Factor 2C; MLC2v: Myosin Light Chain 2 Ventricular; MT: microtissue; MYH7: Myosin Heavy Chain 7; SM: stretchable membrane; NC refers to no change; n.q.: not quantified.

Studies consistently report changes in sarcomere length when comparing stimulated and non-stimulated cardiomyocyte cultures. Studies that have observed a decrease in sarcomere length typically used low-strain stimulation regimes in 2D monolayer systems.^52,89^ In contrast, the 2D monolayer study by Dou *et al*. demonstrated increases in sarcomere length up to 1.91 µm, with a plateau between 15 and 20% strain.^53^ Notably, most studies applying 10% strain reported sarcomere lengths at approximately 1.8 µm or higher, often using a frequency of 1 Hz with brief, continuous stimulation in monolayers or tissues.^53,54,90^ Lu *et al*. reported an even greater sarcomere length of 2.19 µm, in comparison to other studies.^55^ Their work employed a unique actuation regime by progressively increasing strain on cardiac tissues over the course of several weeks, which could contribute to the increased sarcomere length.^55^ Incorporating mechanical strain in cultures can improve sarcomere length and reach more comparable values as observed in adult CMs. In addition, tissue length has been observed to increase with progressive stimulation patterns.

However, few studies have applied active mechanical stimulation to hiPSC- and hESC-derived CMs, making it difficult to determine the optimal duration, stimulation type, or system to promote cardiomyocyte maturation. Nonetheless, structural changes are consistently observed at strain levels of 10–20%. Across various studies, stimulated cardiac monolayers or microtissues generally show improvements compared to unstimulated controls, regardless of the specific actuation parameters. While the extent of improvement varies among systems, there is a general consensus that mechanical stimulation is beneficial. Still, despite these advancements, the maturity observed remains below that of adult CMs.

### 6.2 Protein expression and hypertrophy

A common method to assess the structural maturity of CM monolayers or engineered cardiac tissues is through immunostaining of various maturation markers (*Table 2*). One frequently examined protein is Cx43, a gap junction marker indicative of cell connectivity. However, it remains unclear whether these gap junctions observed in *in vitro* localize to intercalated discs as seen in *in vivo* adult CMs.^96^ Few studies using hESC-CMs^92^ and hiPSC-CMs^89^ report Cx43 at cell–cell contacts irrespective of the systems or actuation regimes used. Although Kroll *et al*. observed Cx43 expression, they did not find any indication of cell polarity, which is typically observed in more mature adult CMs.^89^ A study by Cortes *et al*. reported decreased Cx43 expression in hiPSC-CMs, whereas other studies found no change or an increase under mechanical stimulation.^52^ Notably, Cortes *et al*. applied a lower strain of 2.34% in 2D monolayers, contrasting with higher strains used in other studies.^52^ Additionally, markers like α-actinin and MLC-2v are commonly examined, though variability in marker selection makes direct comparisons challenging.

As CMs mature, they lose proliferative capacity and switch to hypertrophic growth to accommodate increased cardiac workload,^97^ a switch not regularly observed in hiPSC-CMs under *in vitro* conditions. Although multiple factors can influence cardiac hypertrophy,^98^ mechanical stretch has been reported to induce hypertrophy in either hiPSC-CMs or hESC-CMs, with most studies showing elevated *ANP* and *BNP* expression levels. Regardless of the stimulating parameter, reports indicate that cyclic stretching increases CM size and volume.^50,55,90,91^ Compared to adult cells, *MYH6* levels are higher in hiPSC-CMs, though *MYH7* increases with mechanical stimulation as the cells mature. While most studies employ short-term cyclic stretch, Lu *et al*. applied a progressive stimulus for 21 days, during which they observed increased cell volume.^55^ Another sign of maturation is reduced proliferation, indicated by decreased Ki67 expression in 2D monolayers,^90^ although some 3D models have reported an increase in DNA synthesis.^91^ Overall, while mechanical stimulation promotes hypertrophic changes, further investigation is required to fully understand its impact on CM proliferation.

### 6.3 Contractility

Contractile force is a key indicator of *in vitro* cardiac tissue performance. In general, studies employing different platforms and mechanical stimulation regimes have reported an overall 1.4- to 5.1-fold increase in contractile force (*Table 3*), underscoring the critical role of mechanical cues in force development. Similarly, the duration of the stimulation appears to have a positive effect on contractile force development, with the highest reported increases for durations between 14 and 21 days. Moreover, Dou *et al*. demonstrated a direct correlation between applied strain amplitude and increased contractile force in stretchable membranes, with a plateau at 15–20% strain, suggesting cellular overload and a diminished response beyond this threshold.^53^ Progressive stimulation protocols have also proven effective in enhancing contractile force. In a study by Lu *et al*.^55^ EHTs subjected to uniaxial progressive stretch during 21 days reported one of the highest increases in contraction force, which may be attributed to the role of progressive mechanical stimulation in supporting tissue enlargement and physiological hypertrophic growth. Although the only study incorporating dynamic preload in engineered cardiac chambers via pulsatile flow conditioning used neonatal rat cardiomyocytes, it also demonstrated increased contractility, as evidenced by higher pressure generation during contraction.^66^

**Table 3:** Overview of contractile and electrophysiological enhancements in *in vitro* cardiac tissues subjected to active preload stimulation

Ref	Platform	Contractility			Electrophysiology		
		Contraction force	Gene markers	Frank–Starling/FFR	Beating frequency	Ion channels	Other
Chun 2015^88^	SM		↑*TNNT2* ↑*MYL2*				
Kroll 2017^89^	SM				NC	↑ L-type Ca^2 +^ channels	
Cortes 2020^52^	SM		↓ *TNNI1* ↑*TNNI3*				
Dou 2021^53^	SM	↑5%: ↑ 1.27-fold 10% ↑1.40-fold 15%: ↑ 1.65-fold 20%: ↑ 1.68-fold	↑ *MYH7*	FS			
Song 2022^90^	SM		↑ *MYH7/MYH6* ↑ *TTN* ↑ *TNNT2* ↑*TNNI3* ↑*MYL2* ↑ *MYL7*		NC	↑ *CACNA1C* ↑*RYR2*	↑ Relaxation time NC in APD duration
Marsano 2016^51^	BA	↑ 2.38 fold	↑ *MHY7/MYH6*		↑ synchronization		↓ Excitation threshold ↑ Maximum capture rate
Tulloch 2011^91^	BA		↑ *MYH7* ↑ *TNNT2* ↑ *NPPA* ↑ *NPPB*		↑	↑*CACNA1C* ↑*RYR2* ↑*ATP2A2*	
Mihic 2014^50^	BA		↑ *MYH7* ↑ *MEF-2C*		↑	↑*CACNA1C* ↑*SCN5A* ↑*KCNH2* ↑*KCNJ2*	↓ Cycle duration
Zhang 2017^54^	EHT	↑ 1.77 fold		FS		↑*CACNA1C* ↑*SCN5A* ↑*KCNJ2* ↑*ATP2A2* ↑*GJA1* ↑*GJA2*	
Jayne 2021^75^	BA	0.5%: ↑ 1.21-fold 1% ↑1.28-fold 1.5%: ↑ 1.37-fold			↑		
Lu 2021^55^	EHT	↑ 5.1 fold	↑ *MYH7/MYH6* ↓ *NPPB*	FFR			↑ 1.7 fold in upstroke velocity
Kensah 2013^92^	BA	↑ 1.76 fold	↑ *MLC2v*	FS	↑		
Ruan 2015^93^	BA	↑ 5 force	↑*MYH6* ↑ *MYH7* ↑ T*NNT2*	FS	↓		↑ 2.2 fold in upstroke velocity
LaBarge 2019^57^	MT		↑ *MHY7/MYH6* ↑MLC2v				

Upward and downward arrows indicate increases or decreases of the markers, respectively.

APD, action potential duration; ATP2A2, Sarcoplasmic/Endoplasmic Reticulum Calcium ATPase 2 (SERCA2); BA, bioactuators; CACNA1C, Calcium Voltage-Gated Channel Subunit Alpha1C; EHT, engineered heart tissue; FFR, force–frequency relationship; FS, Frank–Starling relationship; GJA1, Gap Junction Alpha-1 (Connexin 43); GJA2, Gap Junction Alpha-2 (Connexin 40); KCNH2, Potassium Voltage-Gated Channel Subfamily H Member 2; KCNJ2, Potassium Inwardly Rectifying Channel Subfamily J Member 2; MEF2-C, Myocyte Enhancer Factor 2C; MLC2V, Myosin Light Chain 2 Ventricular; MT, microtissue. MYH6, Myosin Heavy Chain 6; MYH7, Myosin Heavy Chain 7; MYL2, Myosin Light Chain 2; MYL7, Myosin Light Chain 7; n.q., not quantified; NC, indicates no change; NPPA, Natriuretic Peptide A; NPPB, Natriuretic Peptide B; RYR2, Ryanodine Receptor 2; SCN5A, Sodium Voltage-Gated Channel Alpha Subunit 5; SM, stretchable membrane; TNNI3, Troponin I3, Cardiac Type; TNNT2, Troponin T2, Cardiac Type; TTN, Titin.

The up-regulation of genes associated with cardiac contractility provides a more universal readout across platforms, particularly where direct force measurement is limited. Numerous studies employing active cardiac mechanical stimulation have reported increases in the *MYH7/MYH6* ratio, a widely recognized indicator of cardiomyocyte maturation. For instance, Dou *et al*. reported that *MYH7* was up-regulated in a strain-dependent manner, in agreement with the observed increase in contraction force.^53^ Changes in cardiac troponin isoform expression are also indicative of maturation within the contractile apparatus. A study by Song *et al.*^90^ reported that hiPSC-CMs subjected to two days of continuous cyclic stimulation at 10% strain exhibited increased expression of *TNNT2* and *TNNI3*, which are predominantly found in the adult heart. Similarly, Cortes *et al*.^52^ observed a down-regulation of the foetal isoform *TNNI1* and an up-regulation of *TNNI3* in hiPSC-CMs mechanically stimulated with stretchable membranes during 4 days, despite using a relatively low strain amplitude of 2.34%. Consistent findings have been observed in cardiac microtissues, where spheroids subjected to cyclic stretch at 10% strain for 7 days exhibited increased *MYH7/MYH6 and MYL2/MYL7* ratios, indicating isoform switching towards a more mature CM phenotype.^57^ Interestingly, studies by Tulloch^91^ and Zhang^54^ have shown that continuous cyclic stretch in bioactuators and EHTs platforms led to the up-regulation of *NPPA* and *NPPB* after 3–4 days of stimulation. These genes are not direct maturation markers but do indicate physiological hypertrophy, suggesting that mechanical stimulation not only enhances contractile maturity but also promotes healthy CM growth.

Studying the Frank–Starling relationship in *in vitro* cardiac models remains challenging, partly not only due to the immature phenotype of iPSC-CMs but also due to the lack of experimental setups capable of providing stepwise preload increments with simultaneous contractile force sensing. However, culture platforms adapted for dynamic or progressive preload often enable direct assessment of gradual incremental loads in contractile force response. The Frank–Starling mechanism has been successfully demonstrated across EHTs, engineered cardiac chambers, bioactuators, and stretchable membrane devices. For instance, a study by Ruan *et al*.^93^ demonstrated how stepwise increases in preload within a customized cardiac bioactuator led to enhanced active force production, effectively recapitulating this feature of the native myocardium. Moreover, mechanically stimulated samples exhibited a 2-fold higher force increment upon a 125% stretch with respect to static controls, supporting the adaptative effects of contractile force generation in response to active preload. Similarly, Li *et al*.^56^ reported a linear relationship between the mean pressure increments imposed on engineered cardiac chambers and the developed pressure observed during tissue contraction. Although the samples were not preconditioned with cyclic stimulation, this demonstrates *in vitro* recapitulation of the Frank–Starling mechanism in terms of pump performance readouts.^56^

### 6.4 Electrophysiology

Spontaneous beating is a hallmark of immature foetal CMs or hiPSC-CMs.^99^ While electrical pacing is commonly used to direct the beating frequency of *in vitro* cardiac tissues, synchronization between tissue contraction and the applied stretch frequency has also been observed. In a study by Tulloch *et al*., tissues subjected to cyclic stretch within a bioactuator gradually increased their beating frequency, ultimately synchronizing with the 1 Hz stimulation frequency after three days of continuous stretch.^91^ Mihic *et al*. further reported that constructs under cyclic stretch within a bioactuator exhibited higher spontaneous beating frequencies compared to static controls, nearly matching the applied frequency of 1.25 Hz during a three-day stretching protocol.^50^ In contrast, other studies, particularly those performed with stretchable membranes, have not observed such synchronization or any significant changes in beating frequency under similar mechanical stimulation regimes.^89,90^ This discrepancy could likely be attributed to the fundamental differences between spontaneous electrical depolarization and mechanically induced pacing. Electrical depolarization is characterized by rapid and transient ion fluxes, while mechanical pacing seems to rely on longer-term adaptations involving microtubule-based mechanotransduction, as observed in single-cell CM studies.^76^

A positive force–frequency relationship in response to electrical field stimulation is another feature of cardiomyocyte maturation. Tissues subjected to prolonged electrical stimulation protocols have successfully recapitulated this property *in vitro*.^100^ Interestingly, in a study where EHTs were pre-conditioned with progressive mechanical stretch and then subjected to electrical pacing, a positive force–frequency relationship was observed, suggesting that cardiac tissues exhibit electrophysiological adaptations in response to mechanical stimulation.^55^ This has been further supported by studies demonstrating that cyclic or progressive strain can up-regulate ion channel-related genes. For instance, a study by Mihic *et al*. reported the up-regulation of genes including *CACNA1C*, *SCN5A*, *KCNH2*, *KCNJ2,* and *KCNQ1*, which was accompanied by a decrease in cycle duration from 700 to 500 ms in stimulated samples.^50^ Significant increases in upstroke velocities (from 7.79 V/s to 13.74 V/s),^93^ and prolonged relaxation times (from 700 to 800 ms)^90^ have also been reported upon mechanical stimulation, reflecting the native asymmetry between contraction and relaxation that allows for more efficient ventricular filling. Additionally, studies featuring cyclic stretch of EHTs have displayed more negative resting membrane potentials (from −52 mV to −72 mV) upon mechanical stimulation.^55^ This finding aligns with a study by Marsano *et al*.^51^ which reported a decrease in the electrical stimulation threshold (from 0.86 V to 0.38 V) in mechanically stimulated tissues compared to static controls (*Table 3*).

Taken together, these studies demonstrate overall structural and functional improvements of cardiac tissues in response to dynamic preload. However, the inconsistent effects observed with different stimulation protocols across platforms make it challenging to draw definitive conclusions about the most effective parameters or regimes for achieving optimal cardiomyocyte maturation.

## 7. How close are we to adult-like CMs?

Although active mechanical stimulation has shown to improve hiPSC-CM maturation, the resulting phenotypes reported in current studies still fall short of adult CMs (*Table 4*). For example, while active preload promotes CM elongation similar to the native anisotropy of adult CMs, other key features such as cell size, binucleation, and structural organization remain underdeveloped. Sarcomere lengths typically fall below adult values, and key components like t-tubules, Z-discs, I-bands, and M-bands are either absent or less organized.^72^ Functionally, hiPCS-CMs continue to display spontaneous beating and limited force outputs (0.8–44% of adult tissue).^72^ Electrophysiological parameters, such as action potential upstroke and conduction velocities, are generally an order of magnitude lower than in adult CMs.^109,111^ However, one study by Lu *et al*.,^55^ which applied progressive mechanical loading to EHTs, demonstrated near-adult sarcomere length and force generation, increased mitochondrial content, reduced proliferation, a positive force–frequency relationship, and resting membrane potentials approaching adult physiological values.^101,114^ These promising results highlight the potential of mechanical loading to partially drive hiPSC-CMs to adult structural and functional levels. On a molecular level, signs of maturation through isoform switching of MYH6 to MYH7, TNNI1 to TNNI3, and MYL7 to MYL2 are also evident but remain below adult ratio levels.^101,124^ Titin isoform switching (N2BA to N2B),^124^ a hallmark of adult CMs, has not been reported in the reviewed studies. Metabolically, a few studies have reported early shifts from glycolysis to oxidative metabolism,^54,55^ suggesting a progression towards adult-like fatty acid oxidation. Nevertheless, a more comprehensive characterization, including proteomics, non-coding RNA profiling, and epigenetic landscape mapping could provide additional insights on the current gaps and maturation status of hiPSC-CMs.

**Table 4:** Comparison of key maturation metrics in adult CMs with hiPSC-CMs and values reported in mechanically stimulated cardiac tissues across different platforms

Metric	Adult CMs	hiPSC-CMs	EHTs	Stretchable membranes	Bioactuators
Morphology	Anisotropic 300 µm^2101^	Round, polygonal^102,103^	155 µm^255^	↑ Elongation^52^	↑ Elongation^50,51^
Sarcomere length	2.2 µm^101^	1.6–1.9 µm^103,104^	2.19 µm^55^ 1.8 µm^54^	1.794 µm^90^ 1.911 µm^53^ 1.9 µm^52^ 1.55 µm^89^	1.75 µm^92^
T-tubules	Yes^101^	No^105^	No^54,55^		
Mitochondrial content	30% of cell volume^101^	10%^106^	↑^55^		
Proliferation	No^72^	Limited^107^	↓^90^	↑^51^ ↑^91^ NC^50^	
Force	10–50 mN/mm^272^	0.25 mN/mm^2108^(single cell)	2.96 mN/mm^254^ 11 mN/mm^255^		4.4 mN/mm^292^ 0.08 mN/mm^291^ 1 mN/mm^293^
Upstroke velocity	200–300 V/s^109^	4–50 V/s^103,110^	13.47 V/s^55^		
Conduction velocity	60 cm/s^111^	1.9 cm/s−17 cm/s^112,113^			4.9 cm/s^92^
Resting potential	−85 to −90 mV^114^	−65 mV^115^	−72.4 mV^55^		
Electrophysio-logy genes	↑ CACNA1C,↑ RYR2, ↑ CASQ2, ↑ S100A1, ↑SERCA2↑KCNJ2, ↑ SCN5A^101^	↓KCNJ2,^116^ ↓CASQ2^117,118^ ↓SERCA2^117,118^ ↓SCN5A,^118^ ↓CACNA1C,^118^ ↓RYR2^117,118^	↑CACNA1C ↑SCN5A ↑KCNJ2^54^	↑ CACNA1C^89,90^ ↑RYR2^90^	↑CACNA1C^50,91^ ↑RYR2^91^ ↑SCN5A^50^ ↑KCNJ2^50^
FS	Yes^101^		Yes^54^	Yes^53^	Yes^75,91–93^
FFR	Yes^119^	No^120^	Yes^55^		
MYH7/MYH6 ratio	MYH7 ∼90%; MYH6 ∼10^121^ (Ratio MYH7/MYH6 ∼9)	<1^103^ (early stage)	0.9^54^ 8^55^	1.5^90^	0.27^51^ 5.33^93^ 5.55^92^
TNNI3/TNNI1 ratio	TNNI3 predominant TNNI1 ∼absent^122^	<1^103^ (early stage)		2.67^52^	
MYL2/MYL7 ratio	MYL2 predominant MYL7 ∼absent (for ventricular CMs)^122^	<1^123^ (early stage)	0.9^54^		12^51^
Metabolism	Predominantly fatty acid β-oxidation^101^	Predominantly glycolytic^103^	↑ β-oxidation ↓ anaerobic glycolysis^55^		

Upward and downward arrows indicate increases or decreases of the markers, respectively.

Engineered cardiac chambers and microtissues were excluded from the table due to the limited number of hiPSC-based studies and insufficient quantification of maturation parameters in current reports.

CASQ2, Calsequestrin 2; CACNA1C, Calcium Voltage-Gated Channel Subunit Alpha1C; FFR, force–frequency relationship; FS, Frank–Starling relationship; KCNJ2, Potassium Voltage-Gated Channel Subfamily J Member 2; MYH6, Myosin Heavy Chain 6; MYH7, Myosin Heavy Chain 7; MYL2, Myosin Light Chain 2; TNNI3, MYL7, Myosin Light Chain 7; RYR2, Ryanodine Receptor; Troponin I3; S100A1, S100 Calcium Binding Protein A1; SERCA2a, Sarcoplasmic/Endoplasmic Reticulum Calcium ATPase 2a; SCN5A, Sodium Voltage-Gated Channel Alpha Subunit 5; TNNI1, Troponin I1; NC indicates no change.

To close these gaps, synergistic strategies should be explored. For instance, combining mechanical loading (through active preload and afterload modulation) with hormonal stimulation, fatty acid supplementation, and electrical pacing may more effectively drive structural and metabolic maturation.^125,126^ Co-culture with non-CM cell types, such as macrophages, epicardial, and endocardial cells, could also promote maturation through paracrine signalling.^72^ Other emerging strategies include the use of splicing modulators to induce isoform transitions in key structural and functional proteins, as it has been demonstrated for titin.^125,126^ Similarly, ectopic expression of calcium handling genes^101^ or non-coding RNAs, such as miR-1,^101^ has also been shown to enhance CM maturation. Epigenetic modulators, including histone deacetylase inhibitors or chromatin-modifying proteins, are also being explored to further facilitate the activation of gene programs related to CM maturation.^78,127,128^ Together, these combinatorial strategies could represent a promising path towards generating hiPSC-CMs that more closely resemble adult human CMs.

## 8. Improved modelling of cardiac disease *in vitro* through active mechanical loading

**Figure 5:**
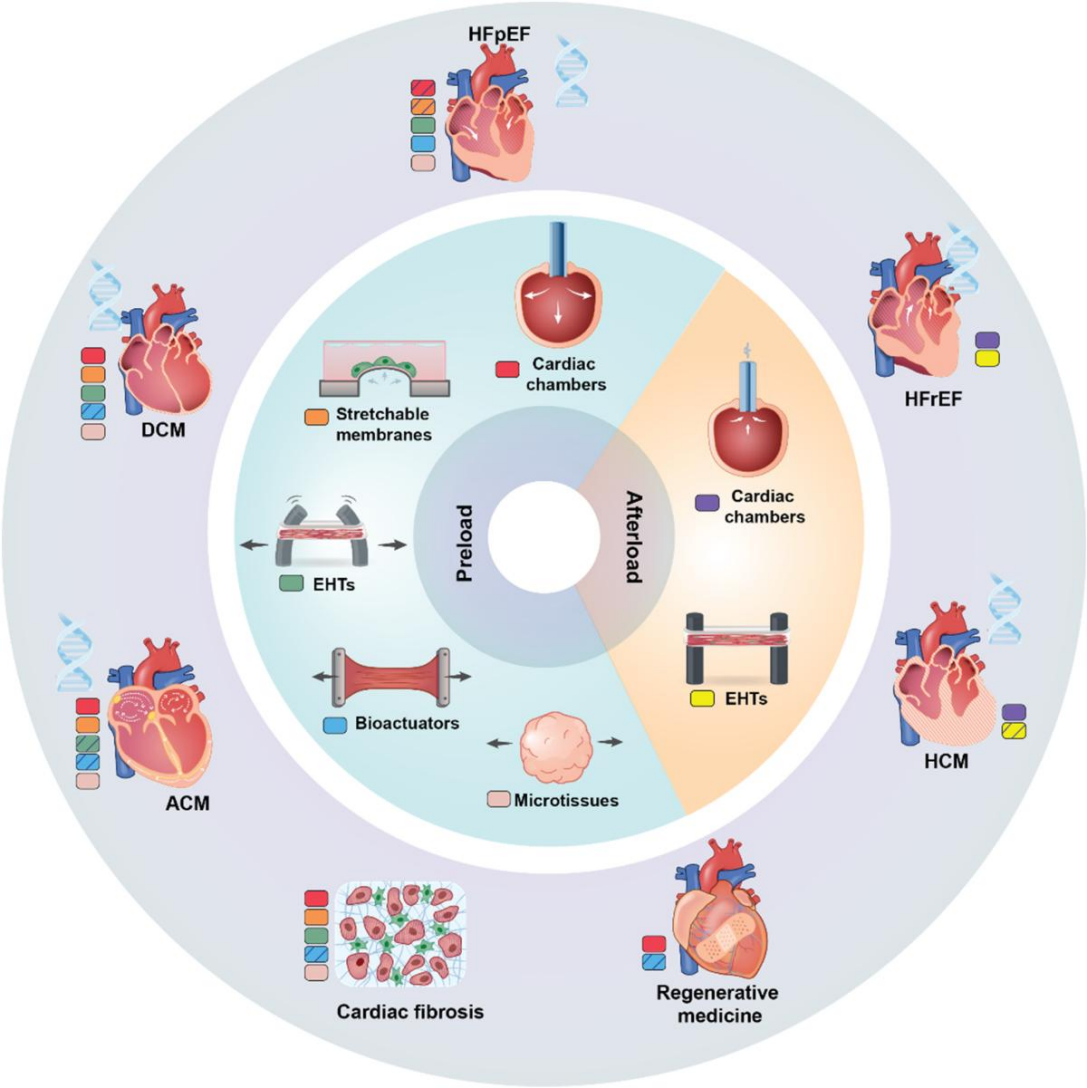
Visual representation of disease modelling applications linked to *in vitro* mechanical loading of cardiac tissues. Incorporation of active preload or afterload in engineered cardiac chambers, stretchable membranes, EHTs, bioactuators, and microtissues has shown promising applications in the modelling of conditions including dilated and hypertrophic cardiomyopathies (DCM, HCM), heart failure with reduced ejection fraction (HFrEF), heart failure with preserved ejection fraction (HFpEF), arrythmogenic cardiomyopathy (ACM), cardiac fibrosis, genetic disorders, and applications in regenerative medicine. The gene symbol indicates that the aetiology of the disease can have a genetic component. Dashed colour-coded boxes indicate key aspects of diseases that have been successfully modelled using each platform. Colour-coded boxes associate each platform with the corresponding possible disease modelling applications.

Platforms mimicking mechanical load dynamics are essential not only for enhancing cardiomyocyte maturation but also for accurately modelling cardiac diseases *in vitro* (*Figure 5*). While the heart naturally adapts to changes in mechanical loading, chronic elevated levels in preload or afterload can lead to pathological remodelling, and the progression of conditions such as genetic cardiomyopathies or post-myocardial infarction injuries is closely associated with preload dynamics. Moreover, integrating active preload into engineered cardiac constructs is critical for advancing future regenerative medicine applications. In the following section, we discuss how current platforms for active preload and afterload can facilitate the modelling of these diseases.

### 8.1 Dilated cardiomyopathy

Dilated cardiomyopathy (DCM), characterized by ventricular enlargement and wall thinning, can arise from genetic mutations in sarcomeric or cytoskeletal proteins, or from chronic exposure to elevated end-diastolic volumes.^129^  *In vitro* mechanical stimulation platforms facilitate the modelling of pathological responses induced by volume overload by modulating the magnitude of the imposed strain. For instance, Rogers *et al*. subjected EHTs to volume-overload conditions through cyclic stretch using a 2% baseline strain and 7% maximum strain at 1 Hz. These reproduced hallmarks of dilated cardiomyopathy, such as an 18% increase in tissue length and up-regulation of desmin expression, compared to tissues subjected to lower strain amplitudes.^130^ Similarly, Chun *et al*., used stretchable membranes to apply cyclic stretch to iPSC-derived CMs from a patient suffering from congenital DCM with unknown aetiology. Unlike healthy controls, the patient-derived cells failed to develop enhanced sarcomere structures upon stimulation, exhibited decreased expression of Cx43 and increased expression of cell–matrix proteins, reflecting maladaptive remodelling in response to mechanical load.^88^

### 8.2 Hypertrophic cardiomyopathy

Hypertrophic cardiomyopathy (HCM) is primarily caused by genetic mutations in sarcomeric proteins, leading to myocardial hypertrophy and, in some cases, to heart failure.^131^ Sustained increases in afterload can worsen HCM or trigger pathological hypertrophy in otherwise healthy myocardium.^132,133^ Incorporating progressively increased afterload in *in vitro* platforms therefore provides a valuable tool both for studying HCM progression, and for modelling pressure-overload-induced pathological hypertrophy. Due to the lack of access to afterload modulation in stretchable membranes and bioactuators, the modelling of HCM is preferably achieved with EHTs or engineered cardiac chambers. For instance, Hirt *et al*., incorporated metal braces to EHTs after 2 weeks of culture to simulate elevated afterload. This induced hallmark features of HCM, including increased CM size, tissue enlargement by 28%, elevated glucose consumption, and activation of foetal gene programmes.^4^ Similarly, Rogers *et al*. modelled increased afterload by maintaining EHTs under isometric culture conditions, hence providing a resistance force sufficient to prevent the tissue shortening upon contraction. This led to tissue widening, up-regulation of collagen-1 deposition and extracellular-matrix remodelling, reflecting a key aspect of pathological hypertrophy.^134^

### 8.3 Arrhythmogenic cardiomyopathy

Arrhythmogenic cardiomyopathy (ACM) is characterized by fibrofatty replacement of myocardial tissue, primarily caused by genetic mutations that disrupt cell-to-cell junctions. This structural disruption leads to cardiac dysfunction and impaired electrical conduction, increasing the risk of ventricular arrythmias and sudden cardiac arrest.^135^ When cell-to-cell adhesions, such as desmosomes, are compromised, cardiac tissues subjected to increased mechanical forces may lose structural cohesion, leading to further malfunctioning.^135^ Hence, combining *in vitro* cyclic stretch platforms and patient-derived CMs offers valuable insights into the role of cardiac load in the progression of ACM. For instance, Ng *et al*. used EHTs made from hiPSC-CMs derived from patients with a desmoplakin mutation and subjected them to normal and increased cyclic diastolic stretch. This resulted in hallmark disease features, including reduced conduction velocities and decreased expression of Cx43.^136^ Similarly, Martewicz *et al*., utilized a bioactuator model incorporating hiPSC-CMs derived from a patient with desmosomal PKP2 mutations subjected to cyclic stretch. The tissues displayed altered transcription profiles linked to pro-fibrotic gene expression programmes, and dysregulation of genes related to cell-to-cell connections.^137^

Another study by Bliley *et al*. investigated the effects of mechanical constraints on disease manifestation using EHT wire constructs. By comparing tissues with a free, unconstrained end to fully anchored tissues, they found that hallmark ACM features, such as impaired desmosome function and contractile abnormalities, were only evident in the unconstrained constructs.^138^ This highlights the critical role of mechanical loads in the manifestation of ACM pathophysiology and the overall potential of genetic disease modelling through patient-derived lines, offering new perspectives for personalized medicine applications.

### 8.4 Heart failure

Heart failure is a clinical condition often arising from cardiomyopathies or myocardial remodelling after myocardial infarction. It can be classified as heart failure with reduced ejection fraction (HFrEF), characterized by impaired systolic contraction and reduced ejection capacity, or heart failure with preserved ejection fraction (HFpEF), where there is proper ventricular ejection, but diastolic filling is impaired due to increased stiffness and reduced compliance of the heart.^139^ The role of mechanical loading in modelling these specific heart failure phenotypes has been less explored *in vitro*. Yeh et al^140^ investigated the effects of aggressive cyclic stretch on cardiac tissues formed on top of stretchable membranes, revealing significant up-regulation of biomarkers associated with acute myocardial infarction (AMI) and HFrEF, such as TnT1, CD105, and SGLT1/2. Interestingly, inhibiting SGLT2 reduced these biomarkers, effectively demonstrating the potential of *in vitro* platforms for testing therapeutic approaches for mechanically induced cardiac damage.

Given their capacity for handling volume changes, engineered cardiac chambers serve as promising tools for studying heart failure. These unique models therefore offer the possibility to replicate the impaired filling mechanics characteristic of HFpEF as well as the impaired ejection function typical of HFrEF. In a recent study by Costa *et al*., engineered cardiac chambers conditioned with TGF-β and endothelin-1 induced HFpEF, as indicated by key hallmarks of the disease, such as increased tissue stiffness and slow relaxation kinetics, without presenting significant decreases in ejection fraction.^141^ Although this study did not incorporate mechanical stimulation, exploring the combined effects of this induced diseased phenotype with increased cyclic preload could provide novel insights into the interplay of mechanical and molecular drivers of heart failure.

### 8.5 Cardiac fibrosis

Cardiac fibrosis is the excessive accumulation of ECM components, primarily collagen, within the heart tissue. This maladaptive remodelling occurs as a response to injury, stress, or disease, such as myocardial infarction, hypertension, or heart failure. By incorporating mechanical loads, *in vitro* models can replicate the mechanical strain cardiac fibroblasts experience *in vivo*, promoting accurate disease modelling. When cardiac fibroblasts are exposed to a physiological range of stimuli (5–10%), they display a quiescent state and have been demonstrated to elicit antifibrotic effects.^61,142^ In contrast, when subjected to higher pathological strain ranges (15–20%), cardiac fibroblasts can undergo a phenotypic transition towards myofibroblasts, with increased expression of α-SMA and ECM components such as COL-1 and fibronectin, compared to unstimulated groups.^61,142,143^ Regardless of the strain incorporated, an increase in ECM protein production and myofibroblast transition is typically observed in models incorporating active loading. Additionally, a study by Visone *et al*. validated a strain-induced fibrosis model using a bioactuator platform and showed the effectiveness of two anti-fibrotic drugs to reduce fibroblast-to-myofibroblast transition in response to cyclic strain.^143^ Furthermore, they utilized their bioactuators to assess the efficacy of novel nanotherapeutics for cardiac fibrosis by directly reprogramming adult human cardiac fibroblasts into induced CMs. These results highlight the importance of screening therapies in relevant tissue models, as cases involving mechanical stimulation demonstrated lower nanotherapeutic efficacy compared to those in a static 3D microenvironment.

### 8.6 Regenerative medicine

In addition to disease modelling, platforms for cardiac mechanical stimulation hold significant potential in regenerative medicine applications.^144^ For instance, a study by Mihic *et al*., developed cardiac grafts and subjected them to cyclic stimulation prior to implanting them on rats with AMI. Remarkably, grafts that were preconditioned with cyclic stretch had better implantation success and enhanced contractility after transplantation.^50^ Another study led by the group of Zimmermann developed engineered heart muscle grafts made from hiPSC-CMs that were mechanically preconditioned with cyclic stretch and implanted in primates with heart failure induced by myocardial infarction. The mechanically preconditioned grafts exhibited enhanced structural and functional maturation that facilitated improved graft integration and remuscularization of scar tissue without inducing arrhythmias, validating the safety and efficacy of these grafts. These promising results led to the approval of a first-in-human clinical trial, where remuscularization after implantation of an engineered heart muscle was observed in a patient with advanced heart failure.^145^

## 9. Discussion

The integration of mechanical stimuli has garnered considerable interest among the different approaches used to improve the structural and functional aspects of hiPSC-CMs. To facilitate this, several *in vitro* platforms, such as bioactuators, stretchable membranes, EHTs, engineered cardiac chambers, and microtissues have been developed to apply dynamic mechanical loads to cardiac constructs, leading to improvements in both tissue architecture and performance. Nonetheless, despite these advancements, the reproducibility of outcomes across studies remains a significant challenge.

The incorporation of active mechanical stimulation involves multiple regimes and parameters that must be considered, such as strain amplitude, frequency, pattern, duty cycle, and total stimulation duration. While there is some understanding regarding the role of certain parameters over functional outcomes, the specific effect of each parameter on tissue performance and maturation remains to be further explored. Investigating the role of individual stimulation parameters and their interplay is crucial to improve our understanding of specific mechanotransduction and cellular responses regarding maturation or disease mechanisms.

However, a key constraint in comprehensive parameter screening lies in the heterogeneity of cell sources and the differentiation stage of cell-types used. The variations in the baseline maturity of cells prior to stimulation differ across studies and contribute to the variability of the responses elicited. Moreover, the cellular composition of *in vitro* cardiac models is often restricted to CMs and cardiac fibroblasts, which does not fully replicate the complex cellular microenvironment as previously discussed. To address these issues, current research aims to further enhance the cellular composition and architecture of engineered cardiac tissues by replicating the epicardial, myocardial, and endocardial layer structures of the heart, and to incorporate additional cell types such as cardiac fibroblasts, immune cells, vasculature, and neural innervation. Although cardiac organoids and self-organized tissues have replicated some of these features,^67,146^ significant hurdles remain in achieving greater complexity and physiological fidelity. In this regard, incorporating mechanical loading into cardiac organoids could further enhance their self-organizing capacity, promoting the formation of more complex and mature tissues, as these cues are crucial for both cardiac development and maturation *in vivo*.

Although each platform for mechanical load modulation offers distinct advantages, their inherent complexity and design differences impede the replicability and comparability of stimulation protocols. Additionally, the lack of standardized functional readouts further complicates cross-study comparisons. The establishment of standardized protocols and regulatory frameworks, such as Translational Organ-on-Chip Platform (TOP) initiatives,^147^ is crucial for enhancing the reproducibility and accessibility of micro-physiological systems and organ-on-a-chip devices. In parallel, high-throughput platforms that enable systematic, independent, and combined screening of stimulation parameters would facilitate discerning specific cellular and tissue responses. Overcoming these challenges is key to optimizing mechanical stimulation protocols for cardiac tissue maturation or disease modelling applications.

Moreover, it would be of interest to define a metric to quantify the total mechanical load applied to the tissue throughout the duration of an experiment. Given the inherent variability in experimental methods and systems, characterizing each setup is essential to ensure accurate force transmission and to understand the induced strain on samples. In combination with a deeper understanding of the role of mechanical stimulation parameters, this would help define a threshold or range of regimes at which mechanical stress promotes beneficial maturation effects vs. those corresponding to mechanically induced pathological responses. Addressing these gaps will expand *in vitro* cardiac tissue engineering applications in disease modelling and therapeutic development.

While active preload modelling has shown promising results, there is still a lack of models for active afterload conditioning. The cardiac response to afterload fluctuations is slower than that to cyclic preload, due to its dependence on slowly varying factors such as arterial pressure and systemic vascular resistance. Despite this complexity, studies clearly show the benefits of incorporating afterload *in vitro* to increase the physiological hypertrophic growth required to observe substantial enhancements in both performance and maturity markers. Moreover, afterload plays a crucial role in modelling cardiac diseases, as pathological conditions such as hypertension, heart failure, and aortic stenosis are inherently linked to increased afterload and its impact on cardiac function.

Bioactuators that integrate cyclic mechanical loading have not only shown promising applications in regenerative medicine but have also shown significant benefits in *ex vivo* studies. The use of bioactuators to maintain *ex vivo* cardiac slices plays a crucial role in preserving their contractility and overall performance, therefore facilitating their utility for research.^148,149^ This technological advancement not only maintains the functional attributes of cardiac slices but also extends their application in drug discovery and disease modelling of the adult human heart.^150^

The integration of mechanical stimulation into *in vitro* cardiac models has become a crucial tool for advancing our understanding of cardiac development, maturation, and disease modelling. By mimicking the biomechanical forces present in the cardiac microenvironment—such as preload and afterload—these models enable the investigation of how mechanical cues influence cellular architecture, sarcomere organization, and functional properties. Active mechanical strain, characterized by parameters such as frequency, amplitude, and duration, has demonstrated significant potential in recapitulating both physiological and pathological conditions. However, standardization of experimental parameters and readouts will be essential to ensure reproducibility and facilitate cross-comparison among studies. Moving forward, leveraging these models holds promise not only for elucidating cardiac biology but also for advancing personalized medicine and accelerating the development of effective therapeutic strategies.

## Data Availability

No new data were generated or analysed in support of this research
